# Comorbidity, Use of Common Medications, and Risk of Early Death in Patients with Localized or Locally Advanced Prostate Cancer

**DOI:** 10.1100/tsw.2011.121

**Published:** 2011-06-09

**Authors:** Carsten Nieder, Astrid Dalhaug, Adam Pawinski, Gro Aandahl, Jan Norum

**Affiliations:** ^1^ Department of Oncology and Palliative Medicine, Nordland Hospital, Bodø, Norway; ^2^ Institute of Clinical Medicine, Faculty of Health Sciences, University of Tromsø, Tromsø, Norway; ^3^ Northern Norway Regional Health Authority, Bodø, Norway

**Keywords:** prostate cancer, comorbidity, statins, NSAID, cardiac disease

## Abstract

In this paper, we analyze predictive factors for early death from comorbidity (defined as death within 3 years from diagnosis and unrelated to prostate cancer) in patients with localized or locally advanced prostate cancer. Such information may guide individually tailored treatment or observation strategies, and help to avoid overtreatment. We retrospectively analyzed baseline parameters including information on comorbidity and medication use among 177 patients (median age at diagnosis 70 years). Actuarial survival analyses were performed. During the first 3 years, two patients (1.1%) died from progressive prostate cancer after they had developed distant metastases. The risk of dying from other causes (3.4%) was numerically higher, although not to a statistically significant degree. Six patients who died from other causes had age-adjusted Charlson comorbidity index (CCI) scores ≥5 (CCI is a sum score where each comorbid condition is assigned with a score depending on the risk of dying associated with this condition). The main comorbidity was cardiovascular disease. The two statistically significant predictive factors were medication use and age-adjusted CCI score ≥5 (univariate analysis). However, medication use was not an independent factor as all patients with age-adjusted CCI score ≥5 also used at least one class of medication. Median survival was 30 months in patients with age-adjusted CCI score ≥5. Prediction of non-prostate cancer death may be important to prevent overtreatment in patients who are more threatened by comorbidity. Our data suggest that simple parameters such as use of medications vs. none, or presence of serious cardiac disease vs. none, are not sufficient, and that age-adjusted CCI scores outperform the other factors included in our analysis.

## INTRODUCTION

Localized and locally advanced prostate cancer (stages T1–T4 N0 M0) is a heterogeneous disease with tremendous variations in prognosis that depend, for example, on serum prostate-specific antigen (PSA) level, Gleason score, and T stage. These combined factors form the basis for the National Comprehensive Cancer Network (NCCN) risk group classification, which stratifies the disease into low-, intermediate-, and high-risk groups[1]. Moreover, the definition of a very low-risk group also includes the number of positive biopsy cores (less than three), whereas a very high-risk group consists of patients with tumors invading the seminal vesicles, bladder, or rectum. Risk group alone is not sufficient to predict overall survival, an outcome parameter that also depends on age and comorbidity[2,3,4,5]. Recent publications have shown that under certain circumstances, death from prostate cancer is less likely than death from other causes, such as diabetes mellitus or cardiovascular disease[6]. For example, D'Amico et al. revealed that death related to diabetes mellitus constituted most (64%) of the deaths of men with prevalent diabetes mellitus and high-risk prostate cancer in their study[7]. Avoiding aggressive treatment in patients who are more threatened by diseases other than prostate cancer is an important goal, as it might prevent unnecessary treatment-related toxicity and cost; especially patients dying from other causes soon after prostate cancer diagnosis might best be managed without prostatectomy or radiotherapy. The present study was performed to identify factors predicting for early death. Because the median follow-up was only slightly more than 3 years, early death was defined as death within 3 years from diagnosis.

## MATERIAL AND METHODS

All patients who presented to the Department of Oncology and Palliative Medicine, Nordland Hospital, Bodø, Norway with newly diagnosed, clinically localized, or locally advanced (T1–T4 N0 M0) prostate cancer for consultation on treatment options during the time period January 1, 2006 to June 30, 2010 were included in this retrospective analysis. Nordland Hospital is the exclusive oncology care provider for the county of Nordland. As previously described[8], this fact and the structure of the Norwegian health care system allows for evaluation of largely unselected patient groups almost comparable to population-based registries, albeit with limited size. None of the patients participated in a formal screening program. All patients were Caucasians, Norwegians born in Norway, and covered by the national public insurance system.

Serum total PSA level was measured with the Siemens ADVIA Centaur® immunoassay system. The T stage was determined by digital rectal examination and transrectal ultrasound performed by the referring urologist. All patients with PSA >20 ng/ml, Gleason score >7, or T3/T4 tumors underwent isotope bone scans. When indicated, additional computed tomography or magnetic resonance (MR) imaging was performed. No evidence of metastatic disease was revealed in any of the patients. After treatment, clinical follow-up and PSA measurements were scheduled every 3 months. All medical records and information on comorbidity, concomitant medications, hospitalization, prostate cancer progression, and cause of death were available in the Hospital's electronic patient record (EPR) system. This EPR system (named DIPS) also contained patient self-reported medication lists, which are routinely collected during admission. As several NSAIDs are available over the counter, self-reported information is important in order to cover all relevant medications correctly. Data on specific dosage or subtype of drug within a given class, e.g., NSAIDs, were not considered.

### Statistical Analysis and Ethics

Patients were selected from the Hospital's EPR system, and the Statistical Package for Social Science (SPSS) version 16.0 was used for the database and statistical analysis, which was performed in January 2011 (last date of follow-up December 31, 2010). For comparison of dichotomous variables, the Chi square test and Fisher's exact test, where applicable, were used and for continuous variables, the Mann-Whitney U-test. Actuarial survival was calculated from histological prostate cancer diagnosis according to the Kaplan-Meier method. Survival curves were compared with the log-rank test. Significance was set to 5%. All tests were carried out two-sided. Median follow-up was 3.3 years (minimum 3 months, maximum 65 months, mean 44.7 months). Seventy-eight patients (44%) had follow-up <3 years. The study was performed as a retrospective analysis of the possible impact of comorbidity on early death. As a quality of care analysis, no approval from the Regional Committees for Medical and Health Research Ethics (REK) was necessary.

## RESULTS

The study population included 177 men with a median age at diagnosis of 70 years. Table 1 shows their clinical characteristics. The majority of patients had high-risk disease (PSA >20 ng/ml and/or Gleason score 8–10 and/or clinical stage ≥T3). Only 58 patients (33%) were diagnosed solely because of elevated PSA levels (typically self-requested measurement). As mentioned, no formal prostate cancer screening was offered to the population in the region. After consultation with both a urologist and a clinical oncologist, 29 patients (16%) chose radical prostatectomy, 65 (37%) radiotherapy (with or without neoadjuvant endocrine treatment), and 83 (47%) endocrine treatment alone as their initial approach. Only a few patients (5.6%) had diabetes mellitus. Table 2 shows additional comorbidity data stratified by age. The main comorbidity was cardiovascular disease. The trend towards increasing comorbidity with age was not statistically significant. Table 3 shows the use of common medications according to age group. Eighty-three percent of patients <60 years did not use statins, NSAIDs, or drugs for cardiac diseases. This figure declined to 43% among patients ≥70 years. The correlation between increased age and drug use was statistically significant ($p$ = 0.006). The most commonly used drugs were statins (34% of all patients, including those who used drug combinations), followed by NSAIDs (27%), and medications for cardiac diseases (27%). The majority of patients used drug combinations, e.g., NSAID plus statin, rather than a single drug.

During the median observation time of 3.3 years, two patients (1.1%) died from progressive prostate cancer after they had developed distant metastases (death after 29 and 32 months, respectively). Both patients had high-risk prostate cancer with a Gleason sum of 9, were at least 70 years old, and received primary endocrine treatment. Their age-adjusted Charlson comorbidity index (CCI) score was 0. The risk of dying from other causes than prostate cancer was higher, although not to a statistically significant degree. Six patients (3.4%) died from intercurrent disease within 6–30 months (Fig. 1). The actuarial rate was 3.7% at 3 years. All six patients had age-adjusted CCI scores ≥5. Table 4 shows the analysis of factors predicting for non–prostate-cancer-related death within 3 years. The two statistically significant factors were medication use and age-adjusted CCI score ≥5. However, medication use was not an independent prognostic factor as all patients with age-adjusted CCI score ≥5 also used at least one class of medication. Fig. 2 shows actuarial non–prostate-cancer-related death rates stratified by comorbidity. Median survival was 30 months in patients with age-adjusted CCI score ≥5.

## DISCUSSION

The present study in a largely unselected patient population (some patients with short survival expectation or low-risk disease might not have been referred to the department), consisting mostly of elderly men with non–screening-detected high-risk prostate cancer, revealed that few patients (4.5%) died within 3 years from diagnosis. D'Amico et al.[7] reported a death rate of 6.8% after 3.9 years (all 5279 men were treated with radiotherapy), which is largely compatible with our results. The majority of deaths in both studies were not related to prostate cancer. Predictive models for non–prostate cancer death might help to prevent overtreatment in patients who are more threatened by comorbidity. Models that are simple and easy

**TABLE 1**
**Baseline Parameters**

| Parameter | All 177 Patients |
|---|---|
| Age at diagnosis, years (median, range) | 70, 48–83 |
| Serum PSA, ng/ml (median, range) | 15.0, 4.1–124 |
| Biopsy Gleason score | |
| 4-5 | 3 (1.7%) |
| 6 | 40 (22.6%) |
| 3+4 | 54 (30.5%) |
| 4+3 | 24 (13.6%) |
| 8 | 28 (15.8%) |
| 9-10 | 28 (15.8%) |
| T stage (AJCC 2003) | |
| T1a or b | 9 (5.1%) |
| T1c | 26 (14.7%) |
| T2a | 27 (15.3%) |
| T2b | 22 (12.4%) |
| T2c | 16 (9.0%) |
| T3a | 63 (35.6%) |
| T3b | 7 (4.0%) |
| T4 | 7 (4.0%) |
| NCCN low risk* | 12 (6.8%) |
| NCCN intermediate risk | 43 (24.3%) |
| NCCN high risk | 122 (68.9%) |
| Diagnosed with urinary symptoms | 119 (67.2%) |
| Diabetes mellitus | 10 (5.6%) |
| Serious cardiac disease** | 29 (16.4%) |
| Age-adjusted CCI score (median, range) | 3, 0–7 |

* Preoperative score in surgically treated patients (41% were reclassified after surgery, typically from intermediate to high risk). T stage and Gleason score in surgically treated patients were also registered preoperatively.

** Myocardial infarction, congestive heart failure, history of coronary bypass surgery or percutaneous coronary intervention, surgery for aortic stenosis.

**TABLE 2**
**Comorbidity Stratified by Age**

| | <60 Years Old (n = 18) | 60–69 Years Old (n = 69) | ≥70 Years Old (n = 90) |
|---|---|---|---|
| Diabetes mellitus | 0 | 2 (2.9%) | 8 (8.9%) |
| Serious cardiac disease | 2 (11.1%) | 9 (13.0%) | 18 (20.0%) |
| Age-adjusted CCI score of 5 or more | 0 | 2 (2.9%) | 9 (10.0%) |

The differences were not statistically significant.

**TABLE 3**
**Use of Different Medications at Diagnosis of Prostate Cancer**

| Medication | <60 Years Old (n = 18) | 60–69 Years Old (n = 69) | ≥70 Years Old (n = 90) |
|---|---|---|---|
| None | 15 (83.3%) | 38 (55.1%) | 39 (43.3%) |
| NSAIDs + statins + cardiac medications* incl. diuretics | 0 | 6 (8.7%) | 4 (4.4%) |
| Only NSAIDs | 0 | 0 | 6 (6.7%) |
| Only statins | 0 | 3 (4.3%) | 3 (3.3%) |
| Only cardiac medications | 1 (5.6%) | 6 (8.7%) | 6 (6.7%) |
| NSAIDs + statins | 2 (11.1%) | 10 (14.5%) | 14 (15.6%) |
| NSAIDs + cardiac medications | 0 | 2 (2.9%) | 4 (4.4%) |
| Statins + cardiac medications | 0 | 4 (5.8%) | 14 (15.6%) |

* Includes, but not limited to, calcium entry blocker, beta receptor blocker, warfarin.

Younger patients were significantly more likely to be non–drug users, $p = 0.006$.

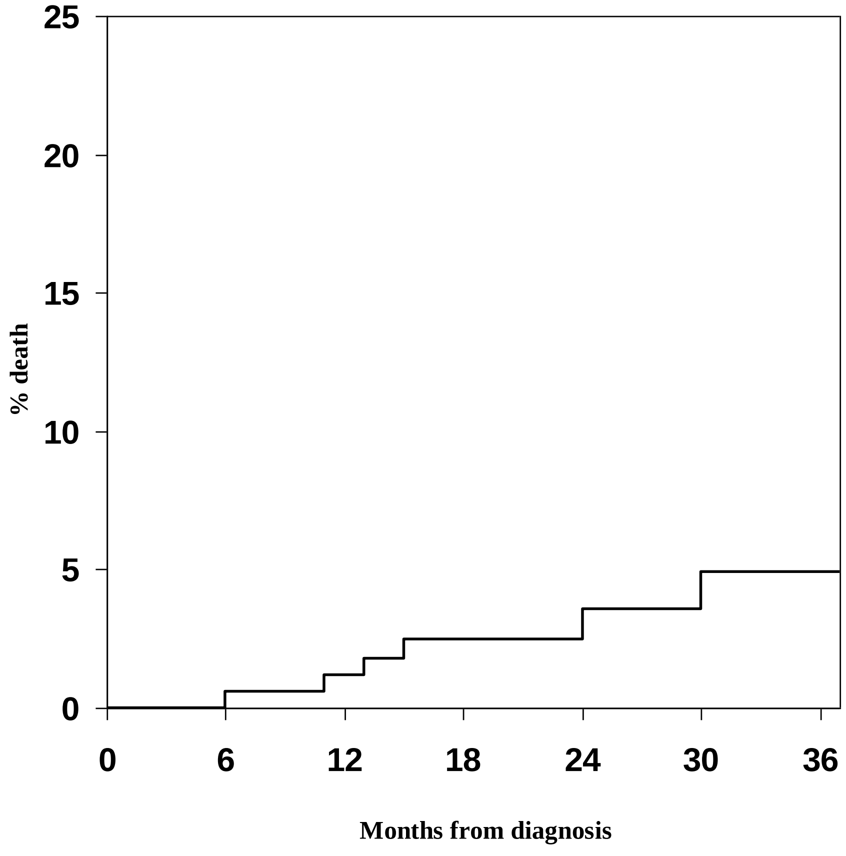

**FIGURE 1.** Actuarial risk of early non–prostate-cancer-related death after diagnosis of localized or locally advanced prostate cancer (Kaplan-Meier analysis, six events).

**TABLE 4**
**Analysis of Factors Predicting for Non–Cancer-Related Death within 3 Years from Prostate Cancer Diagnosis**

| Parameter | Strata | 3-Year Death Rate | *p* Value |
|---|---|---|---|
| Age | <70 years | 2.3% | 0.68 |
| | ≥70 years | 4.4% | |
| Diabetes mellitus | No | 3.0% | 0.30 |
| | Yes | 10.0% | |
| Serious cardiac disease | No | 2.7% | 0.26 |
| | Yes | 6.9% | |
| Medication use | None | 0 | 0.01 |
| | Any | 7.1% | |
| Age-adjusted CCI score | <5 | 0 | 0.008 |
| | ≥5 | 60.6% | |

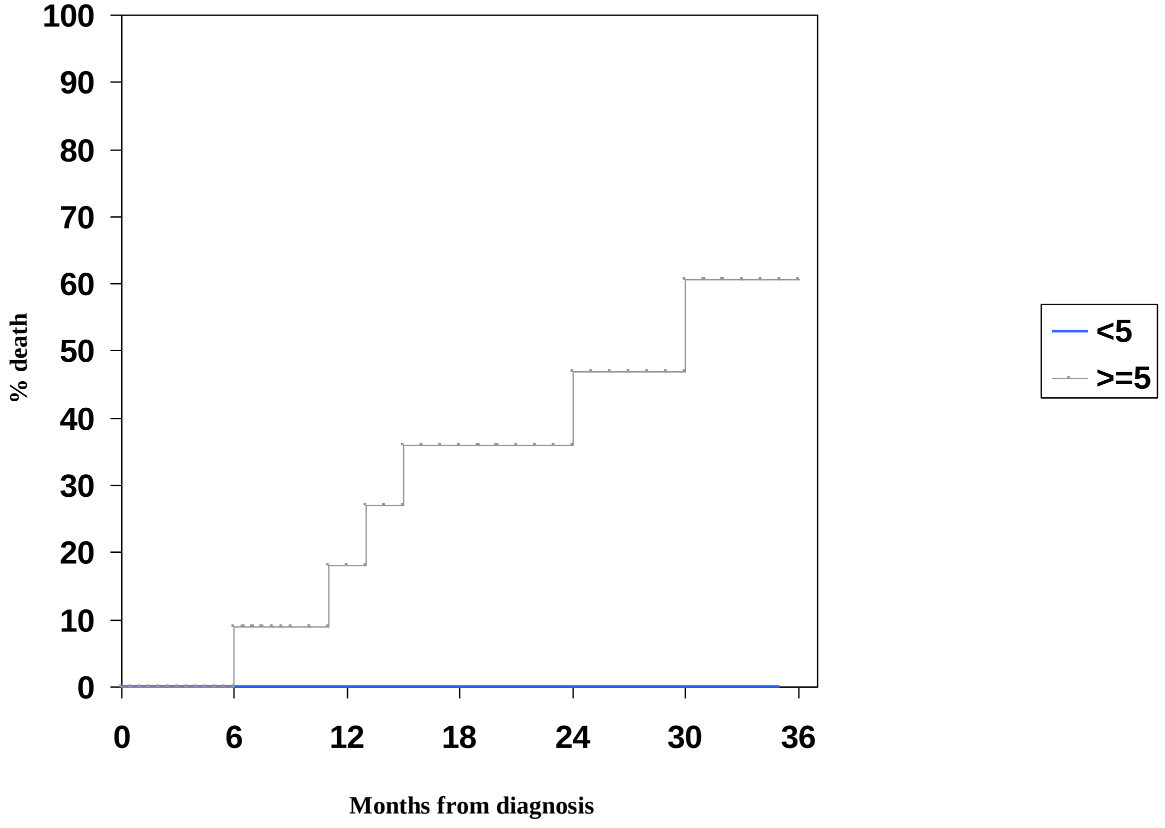

**FIGURE 2.** Actuarial risk of early non–prostate-cancer-related death in patients with age-adjusted CCI score <5 (n = 166) vs. ≥5 (n = 11); Kaplan-Meier analysis, log-rank test, $p = 0.008$.

to administer might gain more clinical acceptance than complex assessment. However, our data suggest that simple parameters such as use of medications vs. none, or presence of serious cardiac disease vs. none, are not sufficient and that age-adjusted CCI scores outperform the other factors included in our analysis. Before discussing other published studies, some comments on design and limitations of our analysis should be given. We arbitrarily decided to choose age-adjusted CCI score, which also has been used in other oncology studies[9,10], rather than other possible tools, and 3 years as the cut-off for early death because the median follow-up was only slightly more than 3 years. Kastner et al. successfully used the CCI score to predict 10-year survival[10] and Alibhai et al.[11] were able to predict 6-year survival with this score. In other words, the CCI score is also a valid tool for long-term survival prediction. The size of our patient population was limited and so was the statistical power of this study. For example, the power to detect a doubling of median survival (from 30 months in 11 patients with age-adjusted CCI score ≥5 to 60 months in 166 patients with age-adjusted CCI score <5) was just under 50% (probability of type I error 0.05). We relied on the Hospital's EPR system for determining whether a patient did or did not use a given medication. No serum drug concentration measurements or pharmacy claims were evaluated. Thus, compliance might have differed from the EPR data. Nevertheless, this analysis provides important insights that might be applicable to other unselected patient populations treated by many community hospitals.

Alibhai et al.[11] compared four comorbidity assessment tools in a study of 345 men who were newly diagnosed with prostate cancer in Ontario, Canada: CCI, Diagnosis Count, Index of Coexistent Disease (ICED), and number of medications. In adjusted models, all four comorbidity indices performed similarly in predicting overall survival. Boulos et al. examined the Chronic Disease Score (CDS), Cumulative Illness Rating Scale (CIRS), Kaplan-Feinstein Index, ICED, and CCI[12]. The differences among indices were not statistically significant. A comorbidity identified at the time of prostate cancer diagnosis was the cause of death in 59% of cases (75% for cardiac or vascular causes). Adult comorbidity evaluation-27 has also been used in other prostate cancer studies[13,14]. No head-to-head comparison of all these tools in a sufficiently large patient group has been published. Therefore, the optimal comorbidity index for use in research remains unclear. In clinical practice, personal experience, accessibility of necessary information, and other less objective factors might guide the choice between these largely interchangeable tools.

As expected, and, in fact, also described previously[15], increasing age correlates with more comorbidity. The prevalence of diabetes mellitus in our patient population (6%) was lower than in other studies (10%[6], 11%[15], 12%[7]). Serious comorbidity, as defined as age-adjusted CCI score ≥5, was detected in 6% of our patients. In a Danish study, 8% of the patients had serious comorbidity (CCI ≥3, not adjusted for age)[16]. In agreement with other studies[17,18], the one from Denmark confirmed comorbidity as a negative prognostic factor for survival. Schymura et al. also used the CCI score in their analysis of 3300 patients treated in 1997 in seven different U.S. states[19]. The risk of death associated with comorbid conditions attenuated with time since diagnosis. Within the first year following diagnosis, the hazard ratio was 2.16 for patients with a comorbidity score of 1 and 6.39 for patients with a score of 2 or more. At 1–3 years post diagnosis, patients with a comorbidity score of 1 were no longer at increased risk of dying compared to patients with no comorbidity, whereas patients with a comorbidity score of 2 or more were still at significantly increased risk, but the hazard ratio was reduced to 2.67. Five-year survival was 87% in patients without comorbidity as compared to 63% in those with two or more comorbidities. Guzzo et al. also reported a hazard ratio of 2.18 for death from other causes than prostate cancer in patients with a CCI score of 2 or more treated with radical prostatectomy[20]. In another study, median survival in patients with prostate cancer and CCI score ≥4 was approximately 3 years[21].

As discussed in many of the articles reviewed here, overtreatment of men with low-risk prostate cancer and men with short life expectancy resulting from concomitant diseases irrespective of NCCN risk group should be minimized in order to avoid unnecessary side effects and cost. Jeldres et al. postulated that life expectancy of candidates for attempted curative therapy of prostate cancer should not be inferior to 10 years[22]. In their population-based cohort, 40% of men in their 70s and 80s who were selected for surgery did not have adequate life expectancy to warrant attempted curative therapy. Even 70% of men who received radiotherapy died before reaching the 10-year survival mark. However, in a minority of patients, such treatment might be necessary to prevent prostate cancer–specific death, and it is therefore important to avoid treatment decisions based on age alone. When trying to improve decision making, it is important to implement comorbidity assessment tools in everyday patient counseling. Given the high probability of death within 3 years in patients with age-adjusted CCI score ≥5 as was observed in our study, aggressive local treatment appears not warranted.
